# The Significance of Histological Typing in the Study of the Epidemiology of Primary Epithelial Lung Tumours: A Study of 466 Cases

**DOI:** 10.1038/bjc.1954.19

**Published:** 1954-06

**Authors:** L. Kreyberg


### THE SIGNIFICANCE OF HISTOLOGICAL TYPING IN THE STUDY OF THE EPIDEMIOLOGY OF PRIMARY EPITHELIAL LUNG TUMOURS: A STUDY OF 466 CASES.

L. KREYBERG.

*From the Institutt for Generell og Eksperimentell Patologi, Universitetet i Oslo.*

Received for publication February 3, 1954.

KREYBERG (1952) in a previous paper analysed a series of one hundred consecutive primary epithelial lung tumours of clinical, mainly surgical origin. The tumours were histologically typed and a brief description was given of the diagnostic criteria used. Those tumours were collected during the years 1948 to 1951.

In the present paper this first series will be designated Series I, and a new material of the same origin, Series II, consisting of 133 cases will be added and the total material used in an analysis of the relationship between histological type and epidemiological aspects. Series I has been reduced to 99 cases as one tumour, originally accepted as a primary, was later shown to be a secondary from a breast carcinoma. Further, because of greater experience, one tumour in this series, designated " malignant adenoma," was revised to a small-cell carcinoma, and another changed from the adenocarcinoma to the salivary gland tumour group. The material in Series II has been collected during the years 1951 to 1953. The largest part has been obtained from the Rikshospital, which receives patients from the whole country. Approximately one-sixth of the material has been placed at my disposal by Professor C. Semb at the Surgical Department III and by Prosector E. Hval at the Pathological Laboratory at Oslo City Hospitals, for which I owe my sincere thanks. A few cases only come from other hospitals.

In addition to the two clinical, two post-mortem series have been used in this analysis, namely, the material of Jacobsen (1953) from Oslo City Hospitals, covering the years 1937 to 1946, and the material of Christiansen (1953) from the Rikshospital, covering the years 1925 to 1952/1. All the tumours of these post-mortem materials have been typed by Kreyberg with no knowledge of the clinical data. The present analysis accordingly is based upon 466 cases.

The total figures of each series are comparatively small, but a few features seem to be significant, and possibly important.

The main differences between the clinical and the post-mortem series are (1) the relatively much higher number of adenocarcinomas, 30 per cent of all cases in the latter against less than 10 per cent in the former, and (2) the lower number of squamous cell carcinomas in the post mortem series. The other histological types are remarkably uniform in their occurrence. A study of the two papers dealing with the post-mortem series reveals a greater number of old persons in these materials, as compared to the clinical group. The significance of these differences will be discussed later.

In Kreyberg's (1952) first paper a comparison was made between the occurrence of the different histological types in the Norwegian material and a similar recent material from the Mayo Clinic. The considerable differences were explained

TABLE I.—*Histological Types of Lung Tumours.*

| Investigator. | Squamous cell carcinoma. Number. | % | Large cell carcinoma. Number. | % | Small cell carcinoma. Number. | % | Adeno-carcinoma. Number. | % | Bronchiolar cell carcinoma. Number. | % | Adenoma salivary gland tumour. Number. | % | Mixed forms, uncertain. Number. | % |
|---|---|---|---|---|---|---|---|---|---|---|---|---|---|---|
| Kreyberg (1952) Series I (99 cases) | 44 | 44·4 | 8 | 8·1 | 18 | 18·2 | 6 | 6·1 | 3 | 3·0 | 16 | 16·2 | 4 | 4·0 |
| Kreyberg Series II (133 cases) | 62 | 46·6 | 6 | 4·5 | 32 | 24·0 | 9 | 6·8 | 5 | 3·8 | 18 | 13·5 | 1 | 0·8 |
| Jakobsen, A. (1937–46) (100 cases) Oslo City Hospital | 19 | 19 | 11 | 11 | 24 | 24 | 30 | 30 | 2 | 2 | 14 | 14 | — | — |
| Christiansen T. (1925–52) (134 cases) The Rikshospital | 39 | 29·1 | 9 | 6·7 | 32 | 23·9 | 39 | 29·1 | 5 | 3·7 | 10 | 7·5 | — | — |
| Foot (1952) Series I, Table IX (123 cases) | 55 | 45 | 15 | 12 | 28 | 23 | 14 | 11 | 5 | 4 | 1 | 1 | 5 | 4 |
| Foot Seriet II, Table X (206 cases) | 115 | 56 | 18 | 9 | 49 | 23 | 20 | 10 | 1 | 0·5 | 0 | — | 3 | 1·5 |
| Foot Series IV, Table XII (330 cases) | 178 | 54·0 | 65 | 20 | 41 | 13 | 37 | 11 | 5 | 1·5 | 2 | 0·7 | 2 | 0·7 |

TABLE II.—*Distribution of Cases of Primary Epithelial Lung Tumours according to Age, Sex and Histological Type of Tumour in 232 Clinical Cases.*

| | 10–19 M. | F. | 20–29 M. | F. | 30–39 M. | F. | 40–49 M. | F. | 50–59 M. | F. | 60–69 M. | F. | 70–79 M. | F. | 80–89 M. | F. | All ages M. | F. |
|---|---|---|---|---|---|---|---|---|---|---|---|---|---|---|---|---|---|---|
| Squamous cell carcinoma | — | — | — | — | 2 | — | 17 | — | 50 | 2 | 28 | — | 7 | — | — | — | 104 | 2 |
| Large cell carcinoma | — | — | — | — | — | — | 3 | — | 3 | 2 | 6 | — | — | — | — | — | 12 | 2 |
| Small cell carcinoma | — | — | — | — | 2 | — | 8 | — | 26 | 2 | 9 | 2 | 1 | — | — | — | 46 | 4 |
| Adenocarcinomas | — | — | — | — | — | — | 1 | 2 | 3 | 2 | 2 | 3 | — | 2 | — | — | 6 | 9 |
| Bronchiolar cell carcinoma | — | — | 2 | — | — | — | — | — | 2 | 2 | 1 | — | — | 1 | — | — | 5 | 3 |
| Adenomas / Salivary gland tumours | — | 1 | 4 | 4 | 4 | 4 | 3 | 4 | 5 | 1 | 1 | 3 | — | — | — | — | 17 | 17 |
| Uncertain | — | — | — | — | — | — | 1 | 1 | 1 | 1 | — | 1 | — | — | — | — | 2 | 3 |
| All types | — | 1 | 6 | 4 | 8 | 4 | 33 | 7 | 90 | 12 | 47 | 9 | 8 | 3 | — | — | 192 | 40 |

TABLE V.—*Number of Cases Occurring in Each Age Group and Each Sex, given in Absolute Figures from the Norwegian Clinical and the Two Post Mortem Materials.*

Quotients (see Table IV and text) have been calculated from the totals of the three materials and for both sexes jointly.

Population background 1950.

| | | | Adenocarciomas. | | | | | Adenomas and salivary gland tumours. | | | | |
|---|---|---|---|---|---|---|---|---|---|---|---|---|
| | | | Materials. | | | All materials. | | Materials. | | | All materials. | |
| | | | K. | C. | J. | Males and Females | | K. | C. | J. | Males and Females | |
| Age. groups. | Sex. | Populations (in thousands). | Number. | Number. | Number. | Number. | Quotient. | Number. | Number. | Number. | Number. | Quotient. |
| 10–19 | M. | 215 | — | — | — | — | — | — | 1 | — | 2 | 0·5 |
| | F. | 206 | — | — | — | — | — | 1 | — | — | | |
| 20–29 | M. | 255 | — | 1 | — | 1 | 0·2 | 4 | — | — | 9 | 1·8 |
| | F. | 245 | — | — | — | | | 4 | — | 1 | | |
| 30–39 | M. | 255 | — | 1 | — | 2 | 0·4 | 4 | 1 | — | 9 | 1·8 |
| | F. | 252 | — | 1 | — | | | 4 | 1 | — | | |
| 40–49 | M. | 220 | 1 | 3 | 3 | 15 | 3·4 | 3 | 2 | 5 | 15 | 3·4 |
| | F. | 227 | 2 | 3 | 3 | | | 4 | 1 | — | | |
| 50–59 | M. | 175 | 3 | 2 | 1 | 20 | 5·5 | 5 | 2 | — | 8 | 2·2 |
| | F. | 186 | 2 | 9 | 3 | | | 1 | — | — | | |
| 60–69 | M. | 112 | 2 | 4 | 3 | 19 | 7·9 | 1 | — | — | 9 | 3·7 |
| | F. | 130 | 3 | 5 | 2 | | | 3 | 1 | 4 | | |
| 70–79 | M. | 68 | — | 2 | 7 | 17 | 11·3 | — | 1 | 1 | 5 | 3·3 |
| | F. | 82 | 2 | 2 | 4 | | | — | 1 | 2 | | |
| 80–89 | M. | 22 | — | — | — | — | — | — | — | — | — | — |
| | F. | 31 | — | — | — | | | | | | | |

mainly as a result of differences in criteria for typing. In the present Table I, three series from Foot (1952) have been included in order to stress this point. The distribution of histological types in this American material is remarkably similar to the Norwegian clinical series, with one exception, the group adenomas and salivary gland tumours. These tumours occur more than ten times as frequently in all the Norwegian series. The cause of the relatively greater number of these tumours may be manifold and will be discussed later. Suffice it to mention at this point that even in America certain investigators have found these tumours rather frequently.

In Table II is presented the distribution of the different histological types as regards age and sex. The material will be referred to in the following discussion.

TABLE III.—*Sex Distribution of the Different Histological Types in the Clinical Material.*

| | | | | | |
|---|---|---|---|---|---|
| Squamous cell ca. | Group I | 104 males | : 2 Females | 52 : 1 | 20 : 1 |
| Large cell ca. | | 12 " | : 2 " | 6 : 1 | |
| Small cell ca. | | 46 .. | : 4 " | 11·5 : 1 | |
| Adenocarcinomas | Group II | 6 " | : 9 " | 0·6 : 1 | 1 : 1 |
| Bronchiolar cell ca. | | 5 " | : 3 " | 1·7 : 1 | |
| Adenomas: sal. gl. t. | | 17 " | : 17 " | 1:1: 1 | |
| Uncertain | | 2 " | : 3 " | | |

In Table III the findings regarding the sex distribution have been summarized. It will be seen that the different histological types in this clinical material form two main groups with markedly different sex ratio. The Group I tumours show a very great preponderance of males, whereas the Group II tumours show a sex ratio of nearly 1:1.

TABLE IV.—*Number of Cases Occurring in Each Age Group and Each Sex, given in Absolute Figures from the Clinical Material.*

Quotient means the relative figure when the cases are seen on the background of the size of the population in 1950.

| Age group. | Sex. | Population (in thousands). | Total lung tumours | | Squamous cell tumours. | | Large cell—small cell. | |
|---|---|---|---|---|---|---|---|---|
| | | | Number. | Quotient. | Number. | Quotient. | Number. | Quotient. |
| 10–19 | M. | 215 | — | — | — | — | — | — |
| | F. | 206 | 1 | 0·5 | — | — | — | — |
| 20–29 | M. | 255 | 6 | 2·4 | — | — | — | — |
| | F. | 245 | 4 | 1·6 | — | — | — | — |
| 30–39 | M. | 255 | 8 | 3·2 | 2 | 0·8 | 2 | 0·8 |
| | F. | 252 | 4 | 1·6 | — | — | — | — |
| 40–49 | M. | 220 | 33 | 15·0 | 17 | 7·7 | 11 | 4·6 |
| | F. | 227 | 7 | 3·0 | — | — | — | — |
| 50–59 | M. | 175 | 90 | 51·4 | 50 | 28·6 | 29 | 16·6 |
| | F. | 186 | 12 | 6·5 | — | — | — | — |
| 60–69 | M. | 112 | 47 | 42·0 | 28 | 25·0 | 15 | 13·4 |
| | F. | 130 | 9 | 6·9 | — | — | — | — |
| 70–79 | M. | 68 | 8 | 11·8 | 7 | 10·3 | 1 | 1·4 |
| | F. | 82 | 3 | 3·6 | — | — | — | — |
| 80–89 | M. | 22 | — | — | — | — | — | — |
| | F. | 31 | — | — | — | — | — | — |

The occurrence of the different tumour types in relation to age has been examined in some detail. In evaluating the age distribution it will be necessary to relate the absolute numbers to the size of the population in the various age groups. In Table IV this relationship is presented for the whole clinical material, males and females separately, for squamous cell carcinomas in males and for large and small cell carcinomas, likewise in males only. The fact that the tumour material has been collected during the years 1948 to 1953, while the population figures refer

FIG. 1.—Age distribution at the time of diagnosis of all lung tumours (L) and the mortality figures for stomach carcinomas (V) in males and females separately. Double logarithmic presentation.

to the year 1950, is of no importance, as a quotient only is aimed at, permitting to present the relationship and to draw corresponding graphs. The figures do not refer to incidences.

Based upon these quotients, in Fig. 1 curves have been drawn on double logarithmic paper, representing the whole material of the clinical series for males and females separately. For comparison one other curve for each sex has been drawn, namely, the corresponding curves for stomach carcinomas, based upon the mortality figures for Norway, 1950. The reason for this special graphic presentation is the fact, recently stressed in this journal by Nordling (1953) that the total cancer incidence in the different countries plotted in this manner shows a straight line.

This is an expression of the usual occurrence for most human carcinomas, breast carcinoma and uterine carcinomas being the most notable exceptions.

As will be seen, the lung tumour curve in both sexes deviates markedly from the curves drawn for comparison. The distinguishing features are mainly : (i) a gradual increase in the lower age groups, (ii) the straight line rise in the age groups 30–39 to 50–59 is broken by a slight decline in the age groups 60–69, and (iii) followed by a very marked decline in the age group 70–79. In females the irregularity of the lung tumour curve is especially striking.

The first tentative conclusion from this graph is, that the lung tumour curve represents a heterogeneous material, that different biological entities are included.

In Fig. 2 two curves have been plotted in the traditional manner, namely, the curves for the relative age frequency of squamous cell carcinomas and for large cell

FIG. 2.—Age distribution of squamous cell carcinomas (A), and large-cell and small-cell carcinomas (B) in males only. Clinical material.

and small cell carcinomas jointly, all in males. The figures are given in Table IV The two curves are nearly identical and they indicate that the Group I tumours biologically actually represent a uniform group, in spite of unquestionable histological differences. It will further be seen that these Group I tumours mainly are responsible for the characteristic features of the lung " cancer " curve in general.

The other lung tumour types have been examined in a similar manner. The adenocarcinomas and the sub-group lung adenomas and salivary gland tumours are, however, comparatively few in number in each of all the series, and accordingly subject to considerable chance fluctuations.

In Table V the figures for these tumours in all the three Norwegian materials are presented. The basic figures are given for each material separately. The quotient has, however, been calculated from the total material, males and females jointly. The few cases which belonged to Christiansen's (1953) as well as Kreyberg's (1952) materials have been included in the latter only. As the sole aim has been to find a picture, as true as possible, of the relative frequency of occurrence of the tumours in the different age groups, such a pooling has been considered permissible. Actually, the pooling probably gives a more accurate picture than the clinical material alone, however great the clinical material might be, because the higher age groups would not be sufficiently represented. This error does not play any role for the Group I tumours, because the number of these tumours in the higher age groups is relatively very small.

In Fig. 3 the quotients have been plotted on the same scale as in Fig. 2, and again the curves show different and characteristic profiles. The adenocarcinomas

FIG. 3.—Age distribution of adenocarcinomas and adenomas and salivary gland tumours in males and females and in the clinical and post-mortem materials together.
——— = Adenocarcinoma.
- - - - - = Adenomas and salivary gland tumours.

increase in number evenly and steadily with the advancing age. The curve belongs to the same pattern as shown for the stomach carcinomas.

The curve for the subgroup lung adenomas and salivary gland tumours show a strikingly even occurrence in the different age groups, indicating that time is not an important factor in the development of these tumours.

Again we have found that the different histological pictures are accompanied by different biological properties.

We have, further, actually found that the curve for all lung tumours represent a heterogeneous material, that lung " cancer " embraces different biological entities. In the general lung "cancer " curve the adenomas and the salivary gland tumours account for the early and slow rise, and the same tumours, and still more the adenocarcinomas, are responsible for the more gradual decline as seen in the higher age groups.

### DISCUSSION.

Squamous cell carcinomas and large and small cell carcinomas, generally regarded as the types of lung tumours connected with special irritants, occur with a very marked preponderance in males, and they show the same peculiar age curve. They very rarely occur before late in the third decade and they show to-day a definite decline after the sixth decade, in striking contrast to most human carcinomas. In spite of different histological pictures, admittedly with transitional forms, it is therefore rational to regard these tumours as a biological entity.

Korteweg (1951) in a penetrating analysis of the lung tumour curve in general, which in his material (from England and Wales) to a great extent was stamped by the Group I tumours, explained the unusual shape of the curve as a result of a new carcinogenic situation, having arisen essentially in this century. Clemmesen, Nielsen and Jensen (1953) further have elaborated this point. The unusual fall in the higher age groups is taken to indicate a new carcinogenic situation in development, and the future stages are forecast by the direction of the curve for the age groups 30–39 and 50–59.

The adenocarcinomas, according to our diagnostic criteria : malignant tumours composed of more or less atypical, secreting or non-secreting, columnar cells, with their nearly equal sex distribution and their increasing frequency with the advancing age, indicate that they are caused by comparatively weak carcinogenic agents, well established in the society, and acting with equal strength in both sexes, at least in large parts of the country.

The small group of bronchiolar cell carcinomas occur in all age groups and with no definite sex preference. They may be caused by unknown agents, hitting at random.

The histological pictures, the equal sex incidence, as well as the appearance with no preference for any age group, together indicate that lung adenomas, benign and malignant, and salivary gland tumours are caused by accidental factors, presumably of developmental origin.

At this stage a few more words about the latter tumours may be justified. Some surprise has been voiced as to the considerable number of adenomas and salivary gland tumours in Norwegian materials.

Firstly, a survey of the lung tumour problem in general in Norway shows that the total number of lung tumours is very moderate, as compared to the figures for England and Wales, the Netherlands, the United States, and even Denmark. If, as assumed and actually found, the recent increase in Norway is caused mainly by the ever larger number of Group I tumours, it follows that the other histological types must show a proportionate decrease in number. This development has been observed in the ordinary columnar cell carcinomas. A decrease in the number of lung adenomas and salivary gland tumours in relation to the sum total is predicted, but it may for a while be less marked in clinical and especially surgical materials, because these tumours represent comparatively favourable clinical cases and are therefore selected.

Secondly, the tumours in question are, actually, observed in considerable numbers also in other materials. Fried (1948) in the Massachusetts General Hospital found 17 adenomas out of 175 cases of bronchogenic neoplasms, and Carlens (1952) reported 8–10 per cent in a Swedish material.

Thirdly, these tumours are most probably present in many other materials without being recognized as such. True columnar cell carcinomas and Group I tumours very rarely occur before the age of 35 years. In most lung cancer statistics some tumours are registered in younger and very young persons. A closer study of the histology of these cases in our own material has revealed a considerable number of lung adenomas as well as a few bronchiolar cell carcinomas. The youngest patient in the records of our Institute is represented by a boy, 12 years old, who suffered from an adenoma, and was reported upon by Harbitz (1937).

Fourthly, these tumours may actually have been seen in the microscope without being accepted as lung adenomas and salivary gland tumours. Some of them have been diagnosed as adenocarcinomas, others as small cell carcinomas, especially if unfavourable preparations have been the base for diagnosis, or if the tumours are infiltrating. Any academic discussion as to the propriety of the designation " malignant adenoma " ought to be silenced by the very important fact, that if the proper criteria are observed and the material typed without clinical information, a group of tumours will emerge, with a sex ratio 1:1, with no accumulation in any age group, and with an important number of cases in young persons. A philological discussion of the proper naming is of secondary interest. To close the eyes to these facts will only lead to the deprivation of some very interesting and important factors in the understanding of the lung tumour problem.

### SUMMARY.

A material of 466 primary epithelial lung tumours have been histologically examined and typed. Each histological type has been analysed, especially as to distribution according to age and sex, and the findings correlated to our additional present knowledge regarding the development of these tumours. The following tentative conclusions have been drawn :

Squamous cell, large cell and small cell carcinomas are found predominantly in males. They do very seldom occur before the age of 35 years and they present a very characteristic and, for human carcinomas, singular age distribution. This age curve as well as the vital statistical evidence indicate that the main part of these tumours are caused by a recently established carcinogenic situation in certain parts of the world, notably the United States of America and Western Europe.

Columnar cell adenocarcinomas appear with little sex difference and they show a steadily increasing frequency with advancing age, a development in conformity with a great many human carcinomas. These tumours are probably caused by comparatively weak carcinogenic influences, evenly distributed over large areas, well established in the society and striking both sexes with equal force.

Bronchiolar cell carcinomas occur with equal frequency in both sexes and strike individuals in all ages. They are presumably caused by yet unknown factors, hitting at random.

Lung adenomas and salivary gland tumours in lungs, bronchi and trachea occur with the same frequency in both sexes and appear in all ages with accumulation in no age group. These factors as well as the histological picture indicate that these tumours are caused by chance developmental factors.

Lung " cancer " is accordingly histologically as well as biologically a heterogeneous group and this fact must be borne in mind when etiological factors are examined.

The present study has been aided by a generous grant from " Tobaksfabrikernes Landsforening av 1901."

I am likewise greatly indebted to Dr. Knut Westlund and Dr. Julie Backer for most valuable assistance during the preparation of the manuscript.

### REFERENCES.

Carlens, E.—(1952) *Acta Un. int. Cancr.*, **8**, 441.
Christiansen, T.—(1953) *Brit. J. Cancer*, **7**, 428.
Clemmesen, J., Nielsen, A., and Jensen, E.—(1953) *Acta Un. int. Cancr.*, **8** Fasc. Spe., 160.
Foot, N. C.—(1952) *Amer. J. Path.*, **28**, 963.
Fried, B. M.—(1948) 'Bronchiogenic Carcinoma and Adenoma.' Baltimore.
Harbitz, F.—(1937) *Norsk Mag. f. Laegev.*, **98**, 1451.
Jakobsen, A.—(1953) *Brit. J. Cancer*, **7**, 423.
Korteweg, R.—(1951) *Ibid.*, **5**, 21.
Kreyberg, L.—(1952) *Ibid.*, **6**, 112.
Nordling, C. O.—(1953) *Ibid.*, **7**, 68.


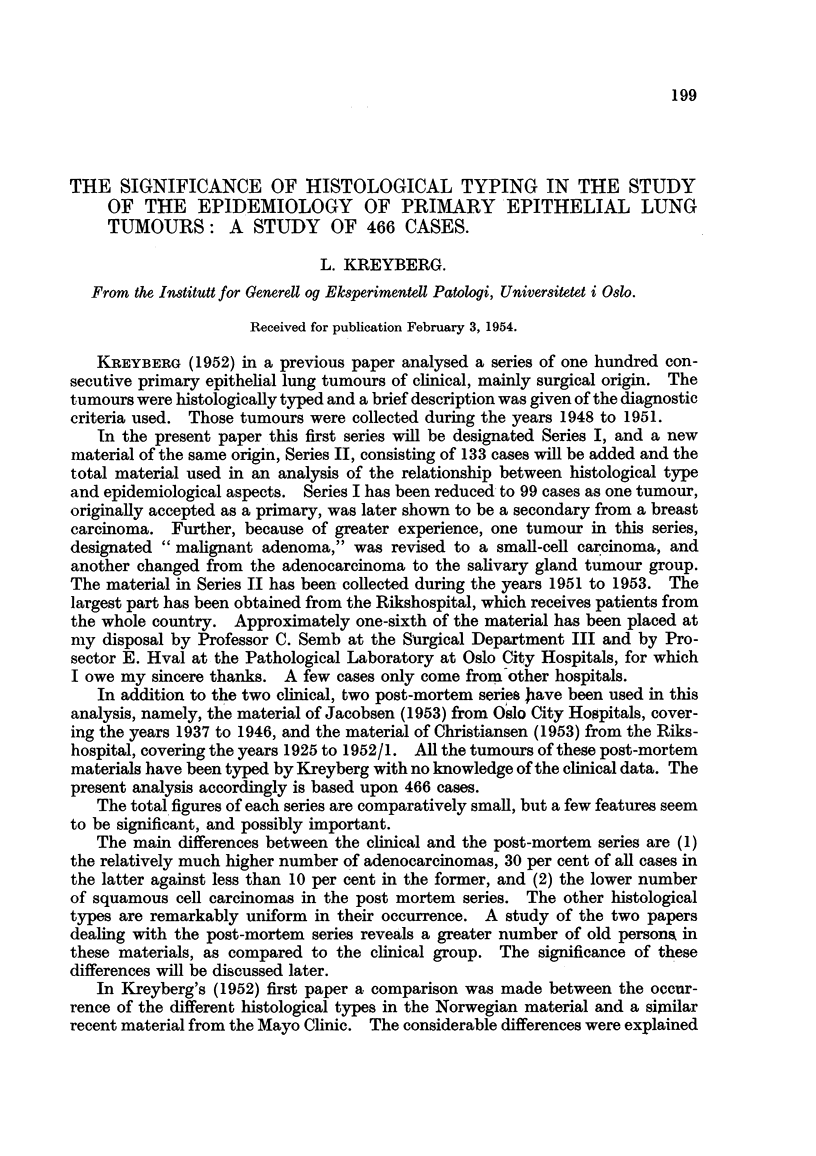

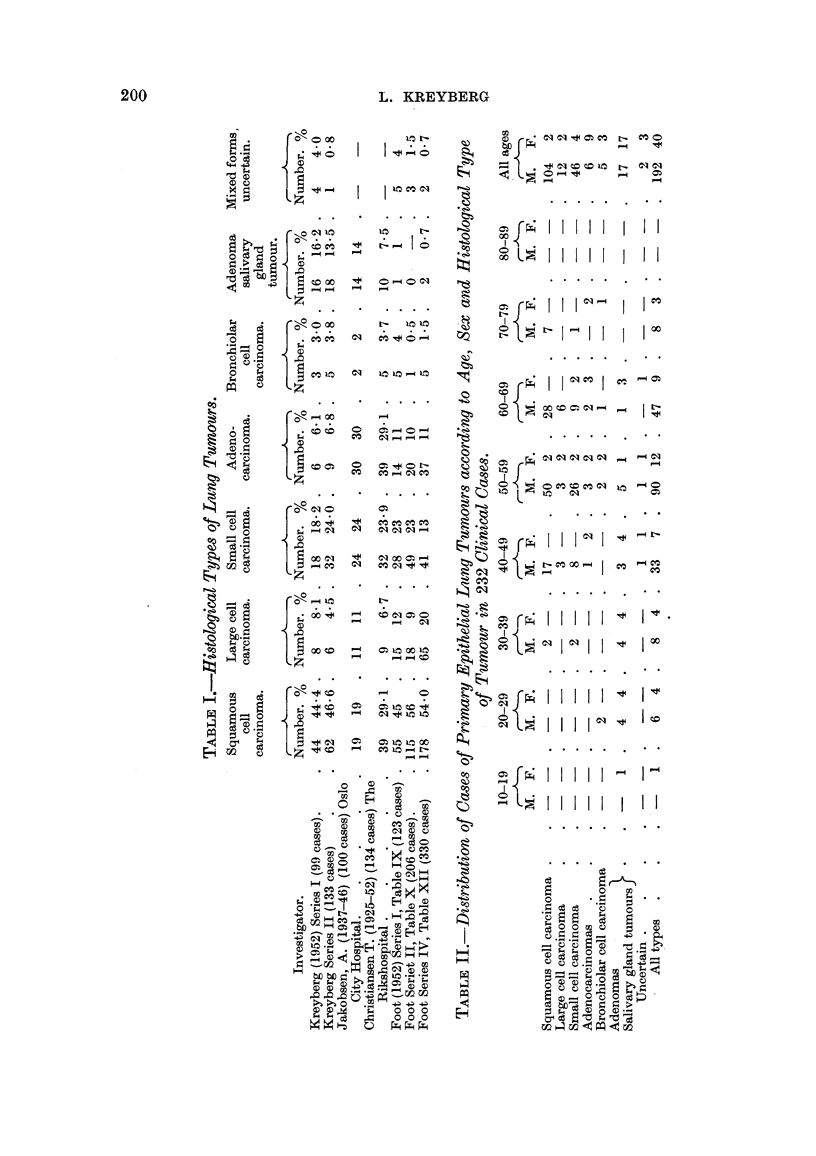

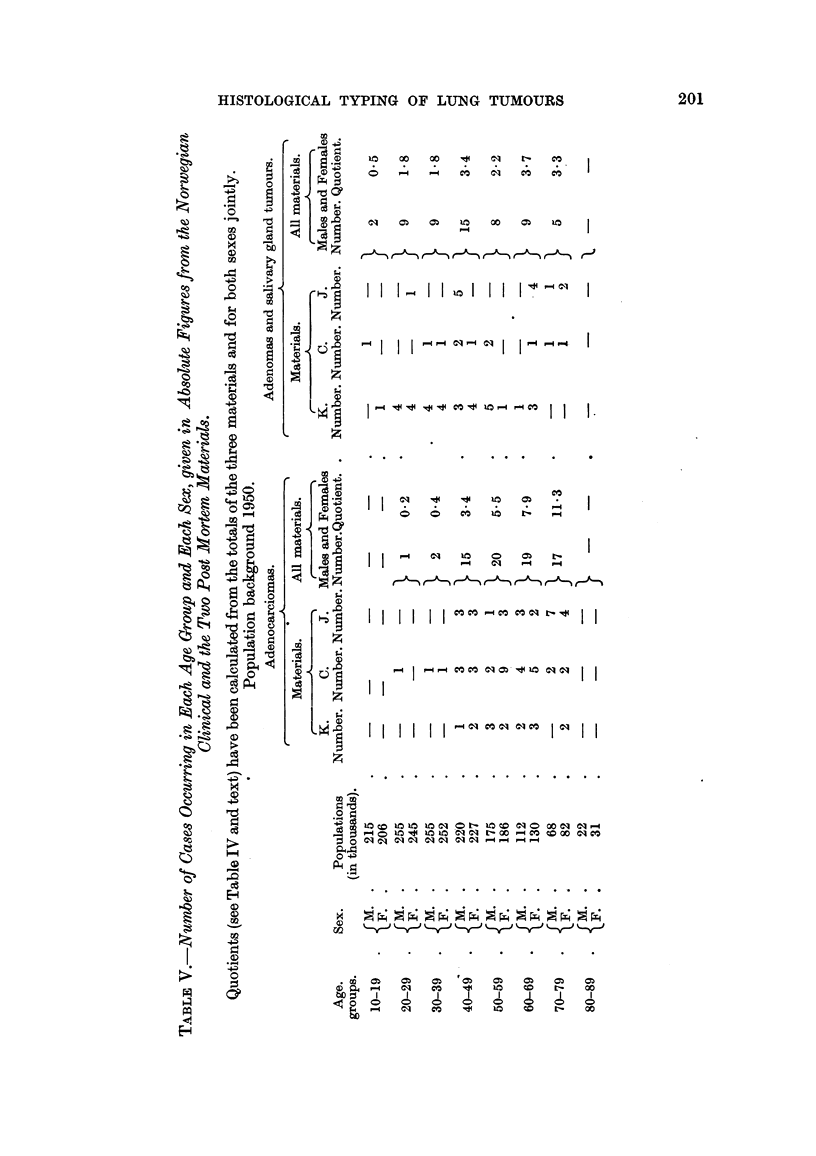

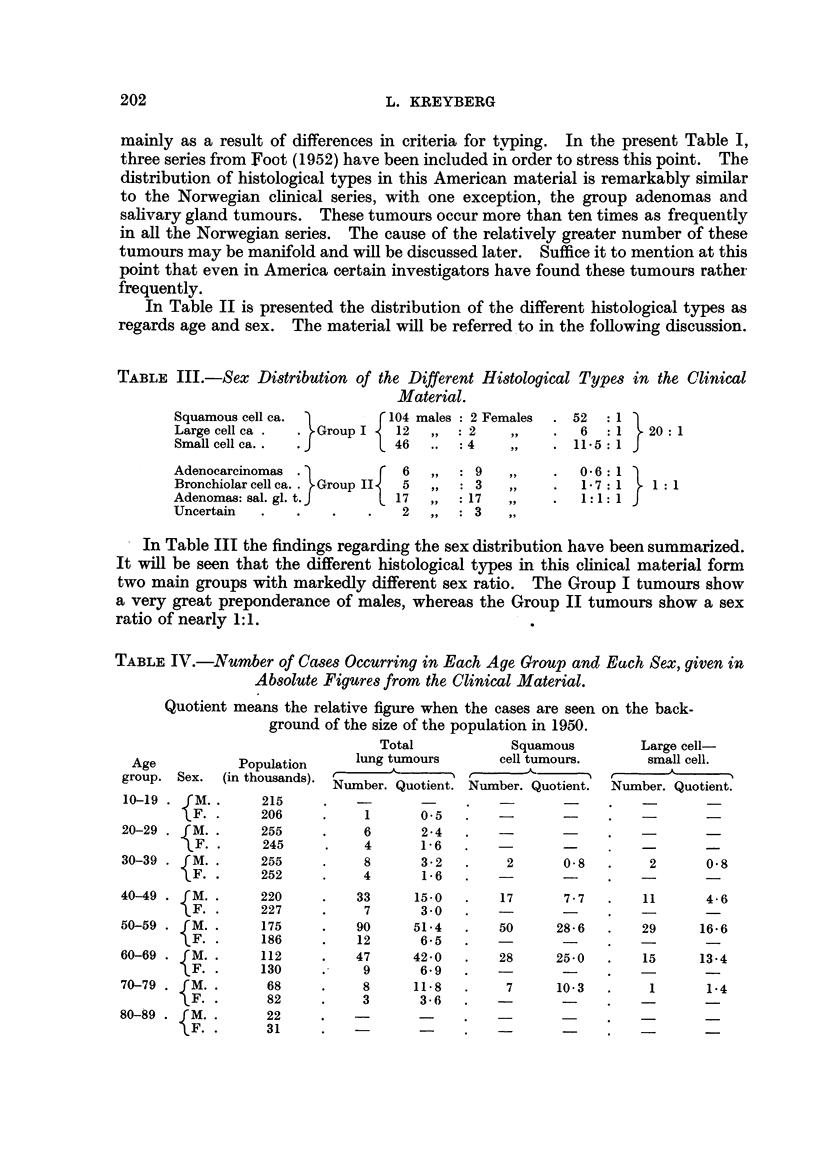

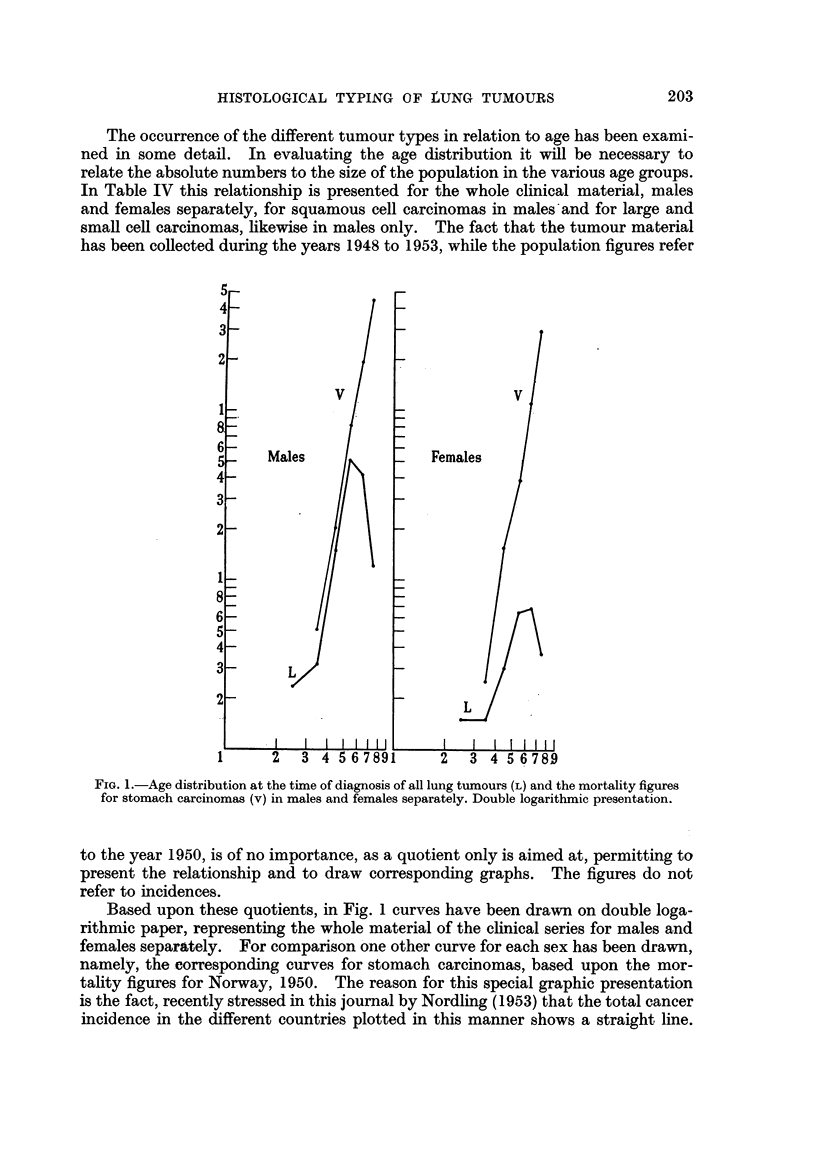

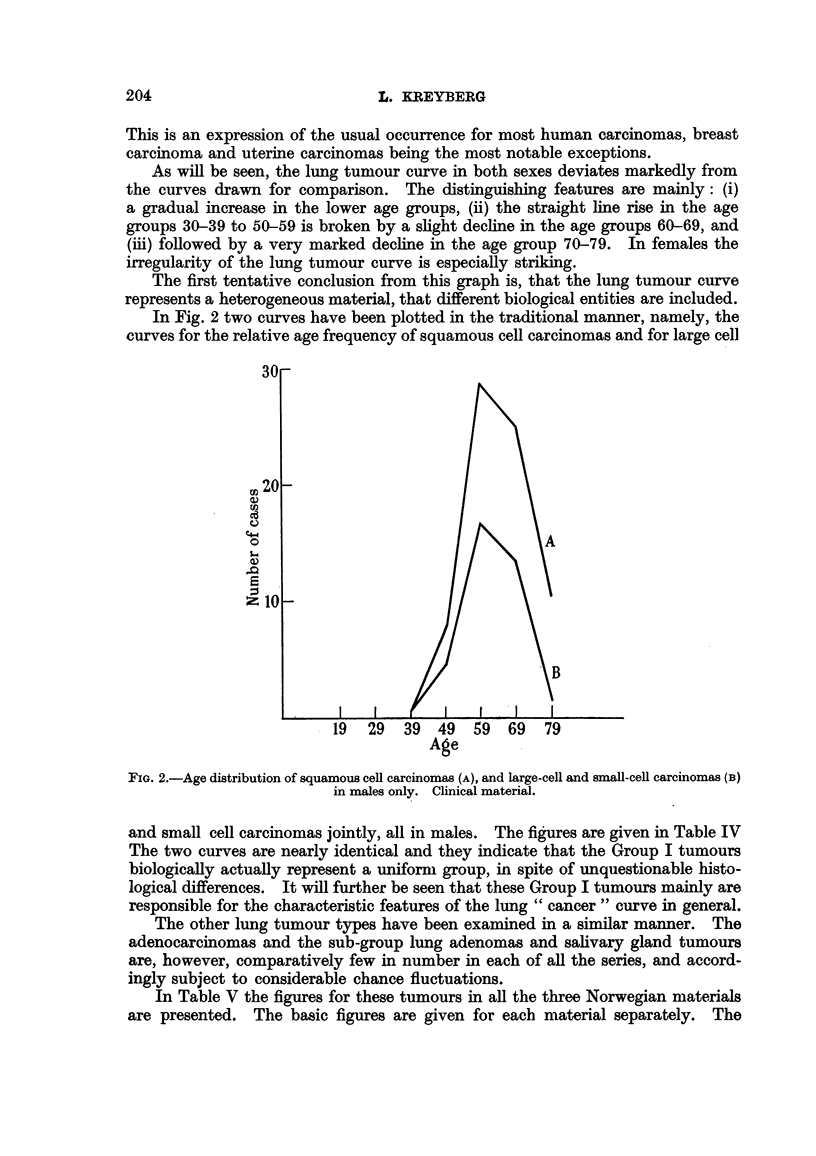

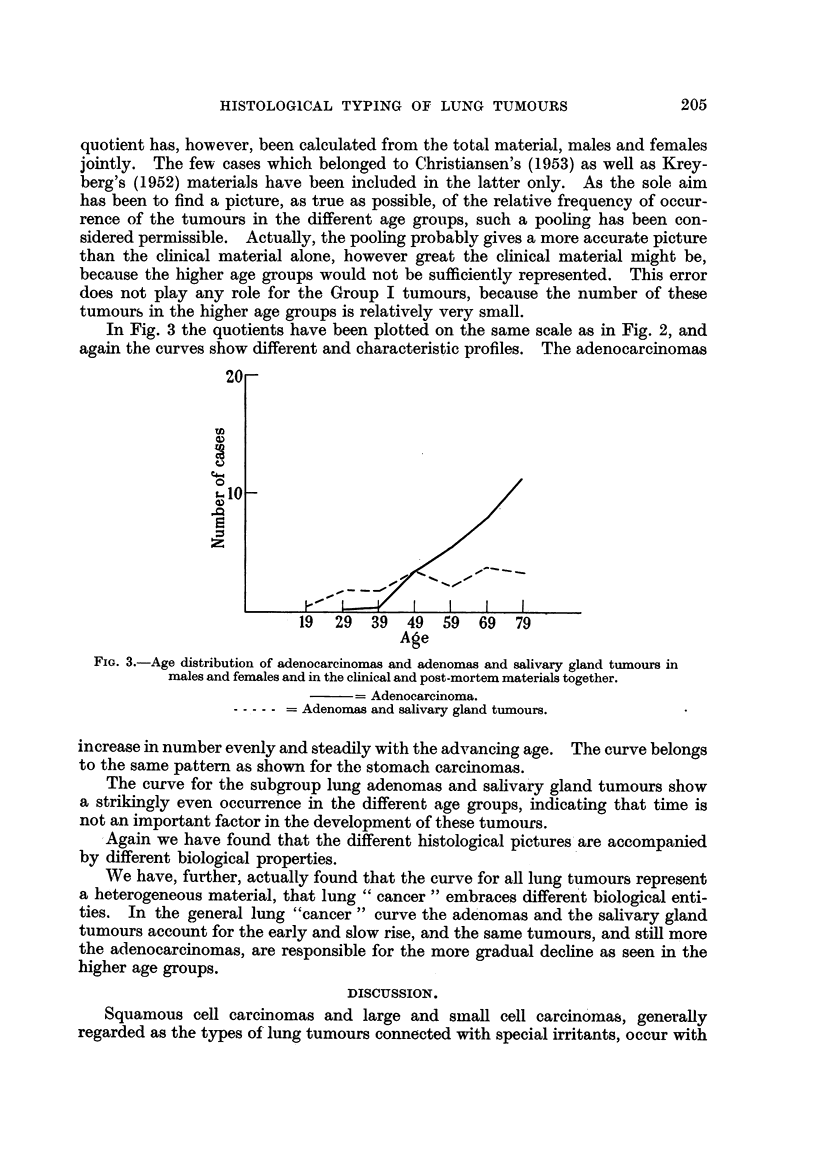

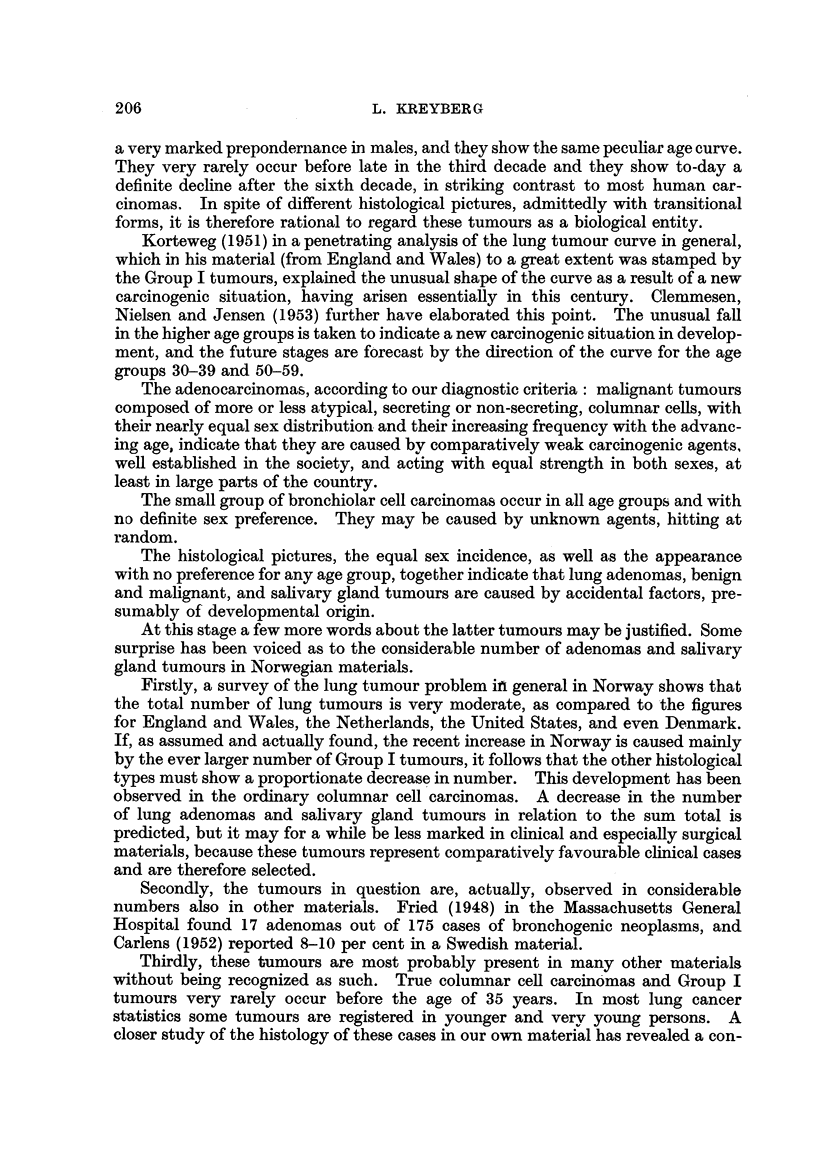

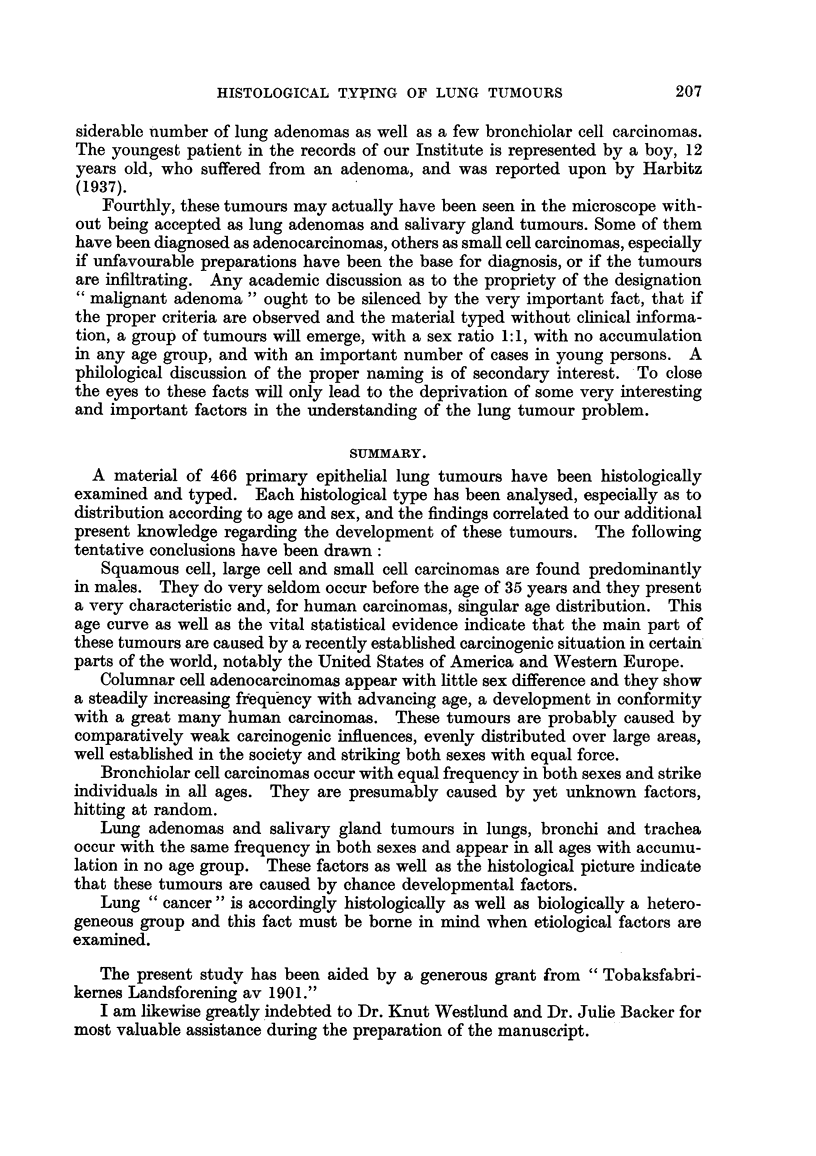

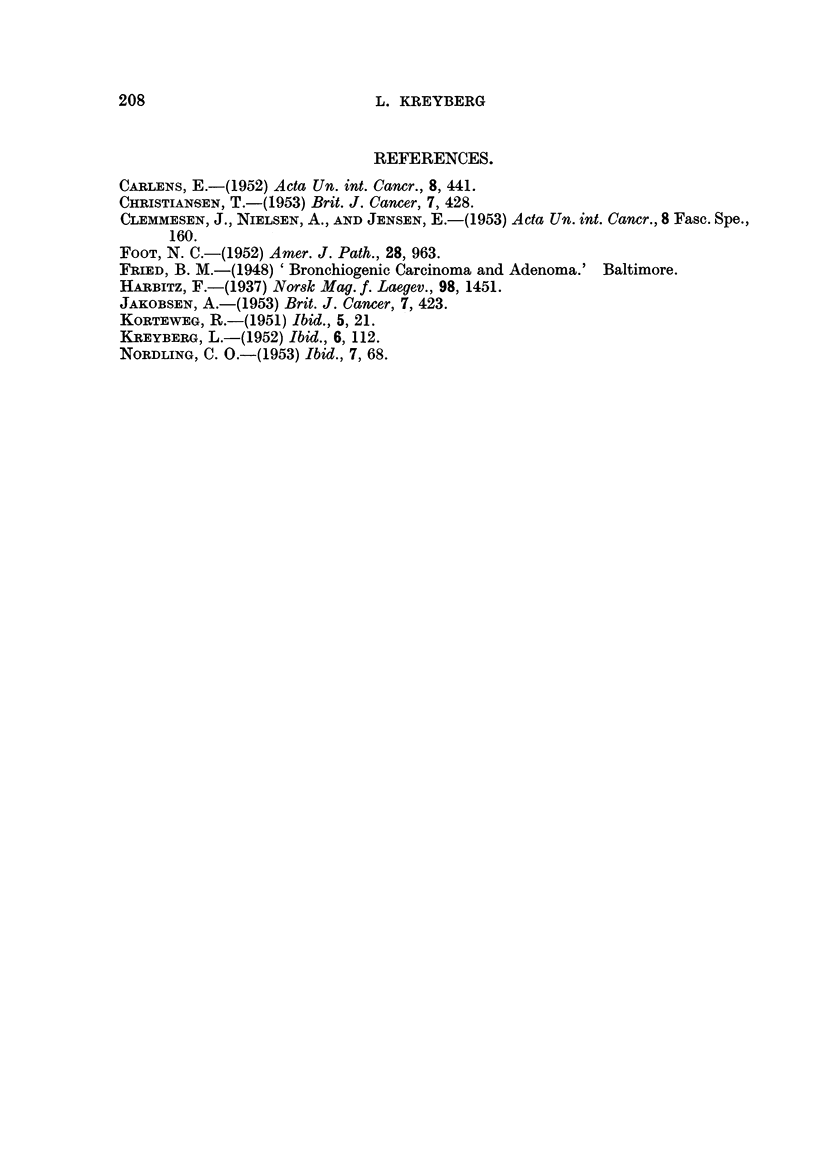


## References

[OCR_00700] BJORK V. O., CARLENS E., CRAFOORD C. (1952). The open closure of the bronchus and the resection of the carina and of the tracheal wall.. J Thorac Surg.

[OCR_00703] CHRISTIANSEN T. (1953). Primary epithelial lung tumours in autopsy material at Rikshospitalet, 1925-52.. Br J Cancer.

[OCR_00707] FOOT N. C. (1952). The identification of types of pulmonary cancer in cytologic smears.. Am J Pathol.

[OCR_00711] JAKOBSEN A. (1953). Primary epithelial lung tumours in post-mortem material from Ullevaal Hospital (Oslo City Hospital).. Br J Cancer.

[OCR_00714] KREYBERG L. (1952). One hundred consecutive primary epithelial lung tumours.. Br J Cancer.

